# Supplementary material for: Stable intracranial imaging of dura mater-engrafted pancreatic islet cells in awake mice
Source: Nat Commun. 2025 Nov 18;16:10047. doi: 10.1038/s41467-025-66057-4 (PMC12627478; doi:10.1038/s41467-025-66057-4)
Supplement: Supplementary file 1 — Supplementary Information [file 41467_2025_66057_MOESM1_ESM.pdf]

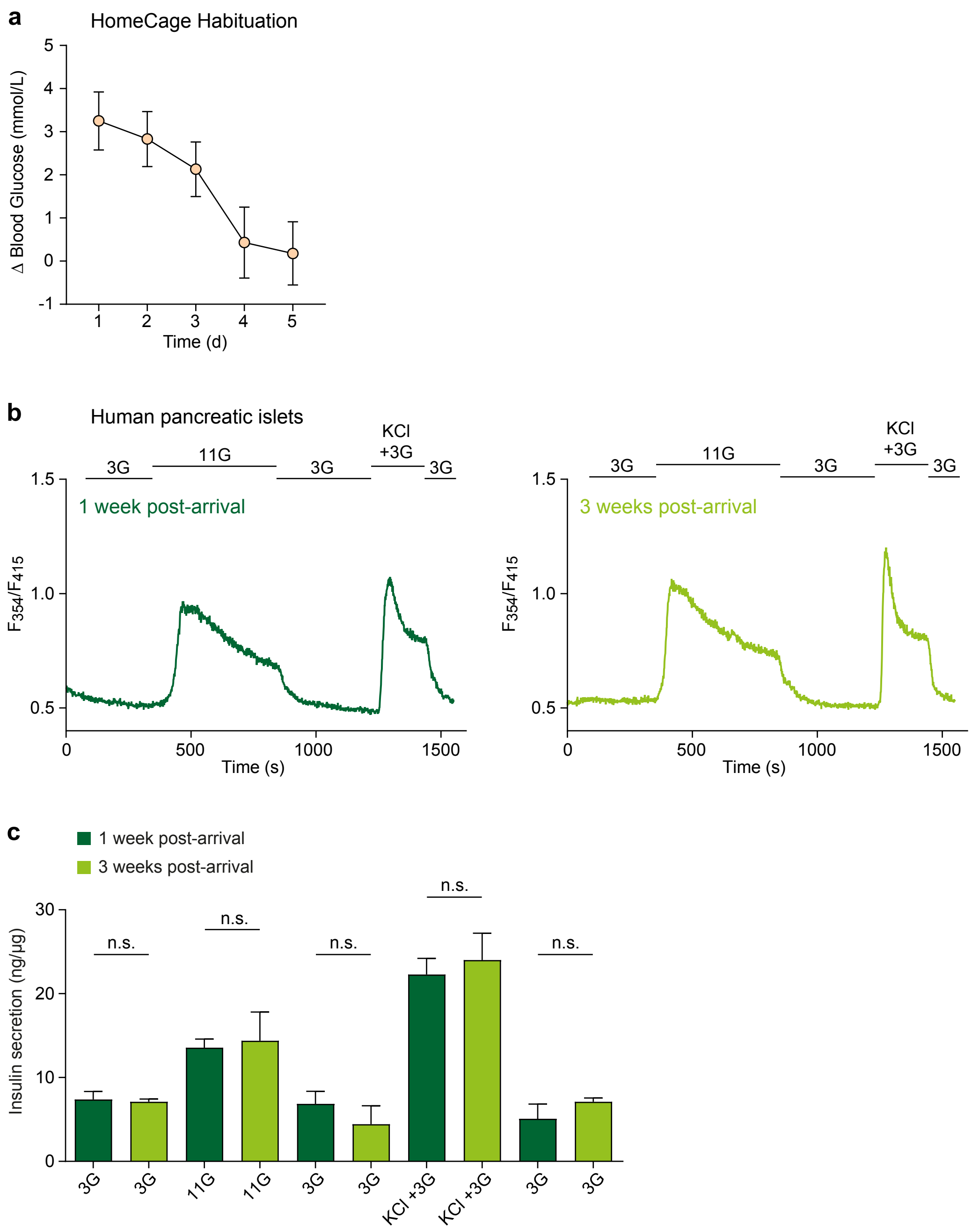

a
HomeCage Habituation
Δ Blood Glucose (mmol/L)
Time (d)
b
Human pancreatic islets
3G
11G
3G
KCl +3G
3G
1 week post-arrival
3 weeks post-arrival
F354/F415
Time (s)
c
1 week post-arrival
3 weeks post-arrival
Insulin secretion (ng/µg)
n.s.
3G
11G
KCl +3G

Supplementary Information

Supplementary Figure 1, Blood glucose during Mobile HomeCage habituation and human islet viability assessment.

(a) Habituation of mice over five consecutive days involved 1 h of restraint per day in the Mobile HomeCage system. Blood glucose was measured immediately before and after each session. Graphs shows change in blood glucose (Δ blood glucose, post–pre). Data are mean ± s.e.m.; n = 4 mice.

(b) Representative Ca^{2+} dynamics of human pancreatic islets. Isolated human islets were assessed one week post-arrival and one week prior to transplantation for glucose-stimulated Ca^{2+} responses using ratiometric Fura-10 imaging in a perfusion chamber. During the ~26 min assay, islets were sequentially exposed to 3 mM glucose (3G), 11 mM glucose (11G), and 25 mM KCl to evoke maximal Ca^{2+} influx. Each time point is representative of n = 3 independent experiments.

(c) Static insulin secretion of human islets in low-volume culture chambers. Eight islets were incubated sequentially in CMRL medium: 1 h in basal glucose (3 mM), 30 min in stimulatory glucose (11 mM), and 30 min in depolarizing KCl (25 mM). Supernatants were collected after each condition and analyzed for insulin content, normalized to total protein. Data are mean ± s.e.m.; n = 5. Statistical analysis, unpaired two-sided *t*-test.
