# Supplementary material for: Stable intracranial imaging of dura mater-engrafted pancreatic islet cells in awake mice
Source: Nat Commun. 2025 Nov 18;16:10047. doi: 10.1038/s41467-025-66057-4 (PMC12627478; doi:10.1038/s41467-025-66057-4)
Supplement: Supplementary file 2 — Reporting Summary [file 41467_2025_66057_MOESM2_ESM.pdf]

nature portfolio

Corresponding author(s): Philip Tröster

Last updated by author(s): Oct 16, 2025

Reporting Summary

Nature Portfolio wishes to improve the reproducibility of the work that we publish. This form provides structure for consistency and transparency in reporting. For further information on Nature Portfolio policies, see our Editorial Policies and the Editorial Policy Checklist.

Statistics

For all statistical analyses, confirm that the following items are present in the figure legend, table legend, main text, or Methods section.

n/a	Confirmed	
☐	☒	The exact sample size (*n*) for each experimental group/condition, given as a discrete number and unit of measurement
☐	☒	A statement on whether measurements were taken from distinct samples or whether the same sample was measured repeatedly
☐	☒	The statistical test(s) used AND whether they are one- or two-sided<br>*Only common tests should be described solely by name; describe more complex techniques in the Methods section.*
☒	☐	A description of all covariates tested
☐	☒	A description of any assumptions or corrections, such as tests of normality and adjustment for multiple comparisons
☐	☒	A full description of the statistical parameters including central tendency (e.g. means) or other basic estimates (e.g. regression coefficient) AND variation (e.g. standard deviation) or associated estimates of uncertainty (e.g. confidence intervals)
☐	☒	For null hypothesis testing, the test statistic (e.g. *F*, *t*, *r*) with confidence intervals, effect sizes, degrees of freedom and *P* value noted<br>*Give P values as exact values whenever suitable.*
☒	☐	For Bayesian analysis, information on the choice of priors and Markov chain Monte Carlo settings
☒	☐	For hierarchical and complex designs, identification of the appropriate level for tests and full reporting of outcomes
☒	☐	Estimates of effect sizes (e.g. Cohen's *d*, Pearson's *r*), indicating how they were calculated

Our web collection on statistics for biologists contains articles on many of the points above.

Software and code

Policy information about availability of computer code

Data collection

Confocal microscopy imaging: LAS AF v2.7.7.12402 (Leica)
ELISA measurements: Wallac EnVision® 2103 Multilabel Plate Reader (PerkinElmer)
ProQuantum immunoassays: QuantStudio5 (appliedbiosystems by Thermo Fisher Scientific)
Blood glucose concentration: Accu-Chek Aviva glucometer (Hoffmann – La Roche)

Data analysis

Image analysis: Fiji/ImageJ (latest version) and Volocity 6.3 (PerkinElmer)
Graphs and statistics analysis: GraphPad Prism v8 (GraphPad Software)
Analysis of β-cell [Ca2+]i dynamics: MATLAB R2017a (Mathworks)

Correlation analyses between the normalized GCaMP3 traces for all selected β-cells in an imaged islet were performed by Pearson's correlation analysis using a custom-made MATLAB script (detailed below). The Cartesian coordinates of the selected β-cells were used to build topographic representations of β-cell pairs based on their correlation strength using a custom-made MATLAB script (detailed below).

Input data: GCaMP3 fluorescence values of the selected β-cells (CaTraces in the code below) with their respective Cartesian coordinates (ptsCaTraces in the code below) were obtained by manually outline individual β-cells GCaMP3 fluorescence signal using Fiji/ImageJ. The intensity changes of GCaMP3 fluorescence was registered over the course of the time series.

```
%% Normalize GCaMP3 signal to the baseline, considered as the average of the lowest 25% of the fluorescence intensity values.
[x,y]=size(CaTraces);
```

```
tend = y;
for tt=1:size(CaTraces,1)
     bug=sort(CaTraces(tt,1:(tend-1)));
     avbl(tt)=mean(bug(1:fix(length(bug)*0.25)));
end
for kk=1:size(avbl,2)
    CaTracesBasal(kk,:)=CaTraces(kk,:)./avbl(kk);
end

%% Perform correlation analyses between the normalized GCaMP3 traces of all β-cells pairs by Pearson’s correlation analysis
[x1,y1]=size(CaTracesBasal);
toffset=1;
twin=240;
ntimepts=floor((y1-toffset)/(twin/2));
    corr_ampl=NaN*ones(ntimepts-1,size(x1,1).^2);

for kk=1:(ntimepts-1)
    tbegin=toffset+(kk-1)*(twin/2);
    tstop=toffset+(kk+1)*(twin/2);
    bug=corrcoef(CaTracesBasal (:,tbegin:tstop)');
    bugB{kk}=corrcoef(CaTracesBasal (:,tbegin:tstop)');
    bug1=tril(bug,-1);
    bug2=bug1(bug1~=0);
    corr{kk}= bug2;
end

%% Build topographic representations of β-cell pairs connected based on their correlation strength. Paired β-cells were interconnected by
color-coded lines corresponding to the following coefficient of correlation range values: blue R = 0.1-0.25, green R = 0.25-0.5, yellow R =
0.5-0.75 and red R = 0.75-1.0.

[totalcells , totaltime]=size(CaTraces);
corrthresh=0.25;
corrthresh2=0.1;
adjmat= bugB{kk};  % Coefficients of correlations matrix you want to perform the network analysis
for i=1:totalcells
    for j=1:totalcells
        if adjmat(i,j)<corrthresh & adjmat(i,j)>corrthresh2
            adjmat(i,j)=1;
        else
            adjmat(i,j)=0;
        end
    end
end

%subplot(2,2,1);

hold on
for i=2:totalcells
    for j=1:i-1
        if adjmat(i,j)==1
            pts1=ptsCaTraces(i,:);
            pts2=ptsCaTraces(j,:);
            plot ([pts1(1) pts2(1)],[pts1(2) pts2(2)],'b');
            set(gca, 'YDir','reverse')
        end
    end
end

hold on
for tt=1:size(ptsCaTraces,1);
  plot(ptsCaTraces(tt,1),ptsCaTraces(tt,2),'b*', 'linewidth',2);
  set(gca, 'YDir','reverse')
end

corrthresh=0.5;
corrthresh2=0.25;
adjmat= bugB{kk};  % Coefficients of correlations matrix you want to perform the network analysis
for i=1:totalcells
    for j=1:totalcells
```

```
        if adjmat(i,j)<corrthresh & adjmat(i,j)>corrthresh2
            adjmat(i,j)=1;
        else
            adjmat(i,j)=0;
        end
    end
end

%subplot(2,2,2);

hold on
for i=2:totalcells
    for j=1:i-1
        if adjmat(i,j)==1
            pts1=ptsCaTraces(i,:);
            pts2=ptsCaTraces(j,:);
            plot ([pts1(1) pts2(1)],[pts1(2) pts2(2)],'g');
            set(gca, 'YDir','reverse')
        end
    end
end

hold on
for tt=1:size(ptsCaTraces,1);
  plot(ptsCaTraces(tt,1),ptsCaTraces(tt,2),'b*', 'linewidth',2);
  set(gca, 'YDir','reverse')
end

corrthresh=0.75;
corrthresh2=0.5;
adjmat= bugB{kk};  % Coefficients of correlations matrix you want to perform the network analysis
for i=1:totalcells
    for j=1:totalcells
        if adjmat(i,j)<corrthresh & adjmat(i,j)>corrthresh2
            adjmat(i,j)=1;
        else
            adjmat(i,j)=0;
        end
    end
end

%subplot(2,2,3);

hold on
for i=2:totalcells
    for j=1:i-1
        if adjmat(i,j)==1
            pts1=ptsCaTraces(i,:);
            pts2=ptsCaTraces(j,:);
            plot ([pts1(1) pts2(1)],[pts1(2) pts2(2)],'y');
            set(gca, 'YDir','reverse')
        end
    end
end

hold on
for tt=1:size(ptsCaTraces,1);
  plot(ptsCaTraces(tt,1),ptsCaTraces(tt,2),'b*', 'linewidth',2);
  set(gca, 'YDir','reverse')
end

corrthresh=0.75;
adjmat= bugB{kk};  % Coefficients of correlations matrix you want to perform the network analysis
for i=1:totalcells
    for j=1:totalcells
        if adjmat(i,j)>corrthresh
            adjmat(i,j)=1;
        else
            adjmat(i,j)=0;
```

```
    end
  end
end

%subplot(2,2,4);

hold on
for i=2:totalcells
  for j=1:i-1
    if adjmat(i,j)==1
      pts1=ptsCaTraces(i,:);
      pts2=ptsCaTraces(j,:);
      plot ([pts1(1) pts2(1)],[pts1(2) pts2(2)],'r');
      set(gca, 'YDir','reverse')
    end
  end
end

hold on
for tt=1:size(ptsCaTraces,1);
 plot(ptsCaTraces(tt,1),ptsCaTraces(tt,2),'b*', 'linewidth',2);
 set(gca, 'YDir','reverse')
end
```

For manuscripts utilizing custom algorithms or software that are central to the research but not yet described in published literature, software must be made available to editors and reviewers. We strongly encourage code deposition in a community repository (e.g. GitHub). See the Nature Portfolio guidelines for submitting code & software for further information.

Data

Policy information about availability of data

All manuscripts must include a data availability statement. This statement should provide the following information, where applicable:

- Accession codes, unique identifiers, or web links for publicly available datasets
- A description of any restrictions on data availability
- For clinical datasets or third party data, please ensure that the statement adheres to our policy

All data are available in the main text or the supplementary information. The data that support the findings of the study are available from the corresponding author (Philip Tröster) upon request and provided by Source Data Excel file.

Research involving human participants, their data, or biological material

Policy information about studies with human participants or human data. See also policy information about sex, gender (identity/presentation), and sexual orientation and race, ethnicity and racism.

Reporting on sex and gender	The term sex was used in the manuscript when referring to human data. Data on the sex of participants was provided by the Nordic Islet Computer System database. Informed consent was obtained from next of kin, and donor identities remained anonymous for ethical and privacy reasons.
Reporting on race, ethnicity, or other socially relevant groupings	n/a
Population characteristics	Patient´s characteristics are as follows: sex, female; age, 70-75 years; BMI = 20-24 kg/m2; HbA1c = 30-40 mmol/mol. This information is included in the manuscript.
Recruitment	Human pancreatic islets were obtained via the Nordic Network for Islet Transplantation from cadaveric organ donors.
Ethics oversight	The protocol for human pancreatic islet procurement and use was approved by the Regional Ethics Review Board in Stockholm (Regionala etikprövningsnämnden i Stockholm; approval number 2006/515-31/3).

Note that full information on the approval of the study protocol must also be provided in the manuscript.

Field-specific reporting

Please select the one below that is the best fit for your research. If you are not sure, read the appropriate sections before making your selection.

☒ Life sciences ☐ Behavioural & social sciences ☐ Ecological, evolutionary & environmental sciences

Life sciences study design

All studies must disclose on these points even when the disclosure is negative.

Sample size	No statistical methods were used to predetermine sample size.
Data exclusions	No data were excluded from the analysis.
Replication	Biochemical assays were performed with a minimum of two independent technical replicates.
Randomization	Donor and recipient animals were randomly assigned.
Blinding	Raw image data requiring manual selection were extracted in a blinded manner prior to data processing and analysis.

Reporting for specific materials, systems and methods

We require information from authors about some types of materials, experimental systems and methods used in many studies. Here, indicate whether each material, system or method listed is relevant to your study. If you are not sure if a list item applies to your research, read the appropriate section before selecting a response.

Materials & experimental systems

n/a	Involved in the study	
☐	☒	Antibodies
☒	☐	Eukaryotic cell lines
☒	☐	Palaeontology and archaeology
☐	☒	Animals and other organisms
☒	☐	Clinical data
☒	☐	Dual use research of concern
☒	☐	Plants

Methods

n/a	Involved in the study	
☒	☐	ChIP-seq
☒	☐	Flow cytometry
☒	☐	MRI-based neuroimaging

Antibodies

Antibodies used	Rat Insulin Alexa Fluor 488-conjugated antibody (R&D Systems, Cat# IC1417G)<br>Guinea pig anti-vesicular acetylcholine transporter (VAChT) (Synaptic Systems, Cat# 139105)<br>Rabbit anti-Tyrosine Hydroxylase (Millipore, Cat# AB152)<br>Goat anti-guinea pig Alexa Fluor 546 (ThermoFisher, Cat# A-11074)<br>Goat anti-rabbit Alexa Fluor 633 (ThermoFisher, Cat# A-21070)
Validation	All antibodies used were validated by the manufacturer. Validation statements can be found in the corresponding manufacturer's website indicated below:<br>Rat Insulin Alexa Fluor 488-conjugated Antibody :<br>https://www.rndsystems.com/products/human-mouse-bovine-insulin-alexa-fluor-488-conjugated-antibody-182410_ic1417g<br>Guinea pig anti-vesicular acetylcholine transporter (VAChT):<br>https://sysy.com/product/139105<br>Rabbit anti-Tyrosine Hydroxylase:<br>https://www.sigmaaldrich.com/SE/en/product/mm/ab152<br>Goat anti-guinea pig Alexa Fluor 546:<br>https://www.thermofisher.com/antibody/product/Goat-anti-Guinea-Pig-IgG-H-L-Secondary-Antibody-Polyclonal/A-11074<br>Goat anti-rabbit Alexa Fluor 633:<br>https://www.thermofisher.com/antibody/product/Goat-anti-Rabbit-IgG-H-L-Cross-Adsorbed-Secondary-Antibody-Polyclonal/A-21070

Animals and other research organisms

Policy information about studies involving animals; ARRIVE guidelines recommended for reporting animal research, and Sex and Gender in Research

Laboratory animals	The mice strains used were C57BL/6J, Ins1Cre:GCaMP3 generated from crossing B6.Cg-Ins1tm1.1(cre)Thor/J with B6.Cg-Gt(ROSA)26Sortm38(CAG-GCaMP3)Hze/J and Rag1-/- (B6.129S7-Rag1tm1Mom/J) mice. For transplantation experiments female mice older than 12 weeks of age and weighting more than 20 grams body weight.
Wild animals	The study did not involve wild animals.

Reporting on sex	Female mice were used as recipients for either mouse or human pancreatic islets transplantation. The choice to only use female mice was to facilitate group housing; no biological rationale influenced this decision.
Field-collected samples	The study did not include samples collected from the field.
Ethics oversight	All animal experiments were approved by the Swedish Animal Welfare Council (Ethical permit: 05480-2023) and conducted in accordance with the “Guide for the Care and Use of Laboratory Animals”, eighth edition (2011).

Note that full information on the approval of the study protocol must also be provided in the manuscript.

Plants

Seed stocks	n/a
Novel plant genotypes	n/a
Authentication	n/a
