# Supplementary material for: Stable intracranial imaging of dura mater-engrafted pancreatic islet cells in awake mice
Source: Nat Commun. 2025 Nov 18;16:10047. doi: 10.1038/s41467-025-66057-4 (PMC12627478; doi:10.1038/s41467-025-66057-4)
Supplement: Supplementary file 3 — Description of Additional Supplementary Files [file 41467_2025_66057_MOESM3_ESM.pdf]

Description of Additional Supplementary Files

Supplementary Movie 1: *Intravital imaging of red blood cell (RBC) flow within intra-islet capillaries of a dura mater-engrafted islet in an awake mouse.*

Supplementary Movie 2: *Intravital imaging of red blood cell (RBC) flow within intra-islet capillaries of a dura mater-engrafted islet in an awake mouse.*

Supplementary Movie 3: *Intravital imaging of red blood cell (RBC) flow within intra-islet capillaries of a dura mater-engrafted islet in an isoflurane-anesthetized mouse.*

Supplementary Movie 4: *Intravital imaging of red blood cell (RBC) flow within intra-islet capillaries of a dura mater-engrafted islet in an isoflurane-anesthetized mouse.*

Supplementary Movie 5: *Confocal time-lapse imaging of β-cell $[Ca^{2+}]_i$ activity in a dura mater-engrafted islet in an awake mouse.*

Supplementary Movie 6: *Confocal time-lapse imaging of β-cell $[Ca^{2+}]_i$ activity in a dura mater-engrafted islet under Hypnorm anesthesia.*

Supplementary Movie 7: *Confocal time-lapse imaging of β-cell $[Ca^{2+}]_i$ activity in a dura mater-engrafted islet under isoflurane anesthesia.*

Supplementary Movie 8: *Time-lapse imaging of 2-NBDG distribution to a dura mater-engrafted islet following subcutaneous glucose administration in an awake mouse.*

Supplementary Movie 9: *Confocal time-lapse imaging of β-cell $[Ca^{2+}]_i$ activity in a dura mater-engrafted islet in an awake mouse without s.c. glucose administration.*

Supplementary Movie 10: *Confocal time-lapse imaging of β-cell $[Ca^{2+}]_i$ activity in a dura mater-engrafted islet in an awake mouse following s.c. glucose administration.*
